# Supplementary material for: How attention simplifies mental representations for planning
Source: eLife. 2026 Jul 9;14:RP108034. doi: 10.7554/eLife.108034 (PMC13349381; doi:10.7554/eLife.108034)
Supplement: Peer review history

# Editor report: eLife Assessment

**DOI:** 10.7554/eLife.108034.3.sa0

**Authors:** Nathan Faivre

**Affiliations:** Centre National de la Recherche Scientifique France

**Keywords:** Convincing, Important

This **important** study utilizes behavioral data and computational modeling to show that spatial properties of visual attention affect human planning. The methodology and statistical analyses are **convincing**, though the way attention is conceptualized and modeled could be refined. The findings of this study will interest cognitive scientists studying attention, perception, and decision-making.

# Referee report: Reviewer #1 (Public review)

**DOI:** 10.7554/eLife.108034.3.sa1

Summary:

This study investigated how visuospatial attention influences the way people build simplified mental representations to support planning and decision-making. Using computational modeling and virtual maze navigation, the authors examined whether spatial proximity and the spatial arrangement of obstacles determine which elements are included in participants' internal models of a task. The study developed and tested an extension of the value-guided construal (VGC) model that incorporates features of spatial attention for selecting simpler task mental representation.

Strengths:

(1) Original Perspective:The study introduces an explicit attentional component to established models of planning, offering an approach that bridges perception, attention, and decision-making.

(2) Methodological Approach:The combination of computational modeling, behavioral data, and eye-tracking provides converging measures to assess the relationship between attention and planning representations.

(3) Cross-validated data:The study relies on the analysis of three separate datasets, two already published and an additional novel one. This allows for cross-validation of the findings and enhances the robustness of the evidence.

(4) Focus on Individual Differences:Reports of how individual variability in attentional "spillover" correlates with the sparsity of task representations and spatial proximity add depth to the analysis.

Appraisal of Aims and Results:

The study sets out to determine how spatial attention shapes the construction of task representations in planning contexts. The authors provide evidence that spatial proximity and arrangement influence which environmental features are incorporated into internal models used for navigation, and that accounting for these effects improves model predictions. There is clear documentation of individual variation, with some participants showing greater attentional spillover and more sparse awareness profiles.

Comments on revised version:

The authors did a great job and I am very happy with the revised manuscript.

# Referee report: Reviewer #2 (Public review)

**DOI:** 10.7554/eLife.108034.3.sa2

Summary:

Castanheira et al. investigate the role of spatial attention for planning during three maze navigation experiments (one new experiment and two existing datasets). Effective planning in complex situations requires the construction of simplified representations of the task at hand. The authors find that these mental representations (as assessed by conscious awareness) of a given stimulus are influenced by (spatially) surrounding stimuli. Individual participants varied in the degree to which attention influenced their task representations, and this attentional effect correlated with the sparsity of representations (as measured by the range of awareness reports across all stimuli). Spatially grouping task-relevant information on either the left or right side of the maze led to mental representations more similar to optimal representations predicted by the value-guided construal (VGC) model - a normative model describing a theoretical approach to simplifying complex task information. Finally, the authors propose an update to this model, incorporating an attentional spotlight component; the revised descriptive model predicts empirical task representations better than the original (normative) VGC model.

Strengths:

The novelty of this study lies in the proposal and investigation of a cognitive mechanism through which a normative model like value-guided construal can enable human planning. After proposing attention as this mechanism, the authors make concrete hypotheses about mismatches between the VGC predictions and real human behavior, which are experimentally validated. Thus, not only does this study describe a possible mechanism for simplification of task information for planning, but the authors also propose a descriptive model, revising VGC to incorporate this attentional component.

A strength of this paper is the variety of investigative approaches: analysis of existing data, novel experiment, and a computational approach to predict experimental findings from a theoretical model. Analyzing pre-existing datasets increases the size of the participant cohort and strengthens the authors' conclusions. Meanwhile, comparing the predictions of the existing normative model and the authors' own refined model is a clever approach to substantiate their claims. In addition, the authors describe several crucial controls, which are key to the interpretability of their results. In particular, the eye tracking results were critical.

In summary, this paper constitutes an important step toward a more complete understanding of the human ability to plan.

Comments on revised version:

I am overall happy with the revision and agree that the authors have addressed most of the comments.

# Referee report: Reviewer #3 (Public review)

**DOI:** 10.7554/eLife.108034.3.sa3

Summary:

The authors build on a recent computational model of planning, the "value-guided construal" framework by Ho et al. (2022), which proposes that people plan by constructing simple models of a task, such as by attending to a subset of obstacles in a maze. They analyze both published experimental data and new experimental data from a task in which participants report attention to objects in mazes. The authors find that attention to objects is affected by spatial proximity to other objects (i.e., attentional overspill) as well as whether relevant objects are lateralized to the same hemifield. To account for these results, the authors propose a "spotlight-VGC" model, in which, after calculating attention scores based on the original VGC model, attention to objects is enhanced based on distance. They find that this model better explains participant responses when objects are lateralized to different hemifields. These results demonstrate complex interactions between filtering of task-relevant information and more classical signatures of attentional selection.

Strengths:

(1) The paper builds on existing modeling work in a novel manner and integrates classic results on attention into the computational framework.

(2) The authors report new and extensive analyses of existing data that shed light on additional sources of systematic variability in responses related to attentional spillover effects

(3) They collect new data using new stimuli in the original paradigm that directly test predictions related to the lateralization of task-relevant information, including eye tracking data that allows them to control for possible confounds.

(4) The extended model (spotlight-VGC) provides a formal account of these new results.

Comments on revised version:

I also agree that the authors addressed our comments and the manuscript is much stronger now.

# Author comment: Author response

**DOI:** 10.7554/eLife.108034.3.sa4

**Authors:** Jason da Silva Castanheira, Christina Chang He, Nicholas Shea, Stephen M Fleming

**Affiliations:** University College London London United Kingdom; University College London London United Kingdom; University of London London United Kingdom; University College London London United Kingdom

The following is the authors’ response to the original reviews.

**Reviewer #1 (Public review):**

Summary:

This study investigated how visuospatial attention influences the way people build simplified mental representations to support planning and decision-making. Using computational modeling and virtual maze navigation, the authors examined whether spatial proximity and the spatial arrangement of obstacles determine which elements are included in participants' internal models of a task. The study developed and tested an extension of the value-guided construal (VGC) model that incorporates features of spatial attention for selecting simpler task mental representation.

Strengths:

(1) Original Perspective:

The study introduces an explicit attentional component to established models of planning, offering an approach that bridges perception, attention, and decisionmaking.

(2) Methodological Approach:

The combination of computational modeling, behavioral data, and eye-tracking provides converging measures to assess the relationship between attention and planning representations.

(3) Cross-validated data:

The study relies on the analysis of three separate datasets, two already published and an additional novel one. This allows for cross-validation of the findings and enhances the robustness of the evidence.

(4) Focus on Individual Differences:

Reports of how individual variability in attentional "spillover" correlates with the sparsity of task representations and spatial proximity add depth to the analysis.

We thank the Reviewer for their overall positive assessment of our work and their helpful comments. We have addressed each point below.

Weaknesses:

(1) Clarity of the VGC model and behavioral task:

The exposition of the VGC model lacks sufficient detail for non-expert readers. It is not clear how this model infers which maze obstacles are relevant or irrelevant for planning, nor how the maze tasks specifically operationalize "planning" versus other cognitive processes.

The method for classifying obstacles as relevant or irrelevant to the task and connecting metacognitive awareness (i.e., participants' reports of noticing obstacles) to attentional capture is not well justified. The rationale for why awareness serves as a valid attention proxy, as opposed to behavioral or neurophysiological markers, should be clearer.

We thank the reviewer for urging further clarity here. Our work builds closely on the previous maze navigation paradigm and VGC model developed and reported by Ho et al. Nature (2022). We directly adopted variants of their maze stimuli, computational model and obstacle awareness measures, and married these with an investigation of the role of visuospatial attention. We agree that it would be useful for the reader to have a more in-depth description of the paradigm and model, and how it operationalises planning, without needing to refer back to the original Ho et al. paper. We have now added additional explanatory sections to the Introduction and Methods as follows:

On page 4:

“One elegant approach to forming such a simplified representation is to adaptively select the granularity of information required to complete the task (Ho et al., 2022), known as value-guided construal (VGC). Unlike previous accounts, which model human planning as a search over all items (e.g.., tube lines), the VGC model predicts that a cognitively limited decision-maker selects a manageable subset of information over which to plan— i.e., a task representation—balancing utility and complexity (Ho et al., 2022). In our example, the VGC algorithm would plan over a few relevant tube lines rather than planning over all possible stations. To select the representation that achieves the best balance between utility and complexity, the model searches across all possible combinations of tube lines, computing the value (i.e., the plan’s utility minus its cost) of each representation for planning a specific journey. The algorithm then selects the representation with the highest value, which ensures that an ideal observer selects a representation which only includes the items (i.e., tube lines) that lead to successful planning while excluding as many items as possible to keep the plan as simple as possible. For our purposes, items included in the representation are considered taskrelevant, while items that are not represented are considered task-irrelevant. This algorithm, therefore, provides a normative standard of an efficient plan to which we can compare people’s actual plans.”

On page 6:

“We operationalized planning using a maze navigation paradigm, akin to our tube-related example, where participants were required to plan a route through the maze, avoiding obstacles that blocked their path. Obstacles predicted by the sVGC model to be included in the representation were considered task-relevant.”

“At the end of every trial, participants reported their awareness of specific obstacles (see Methods for details). The level of awareness reported for different obstacles provides a read-out of what features of the environment individuals were subjectively representing while solving a particular maze. While other markers of attention and awareness (for instance, behavioural or neurophysiological variables) could also be used, here we focused on direct awareness reports in order to relate our findings both to those of Ho and colleagues and to the subjective awareness reports used in consciousness science (e.g. the Perceptual Awareness Scale (Barnett et al., 2024; Overgaard & Sandberg, 2021; Ramsøy & Overgaard, 2004; Samaha et al., 2015)). Participants were instructed to maintain central fixation while planning (see dataset dSC 1), in line with previous empirical work using this task (Ho et al., 2022).”

To visualize our effects, we binarized the predictions of the sVGC model such that obstacles with a marginalized probability greater than 0.5 were considered taskrelevant, while other obstacles were considered task-irrelevant (e.g., Figure 2b). We now clarify this point in the caption of Figure 2.

(2) Attention framework:

The account of attention is largely limited to the "spotlight" model. When solving a maze, participants trace the correct trail, following it mentally with their overt or covert attention. In this perspective, relevant concepts are also rooted in attention literature pertaining to object-based attention using tasks like curve tracing (e.g., Pooresmaeili & Roelfsema, 2014) and to mental maze solving (e.g., Wong & Scholl, 2024), which may be highly relevant and add nuance to the current work. This view of attention may be more pertinent to the task than models of simultaneously tracking multiple objects cited here. Prior work (notably from the Roelfsema group) indicates that attentional engagement in curve-tracing tasks may be a continuous, bottom-up process that progressively spreads along a trajectory, in time and space, rather than a "spotlight" that simply travels along the path. The spread of attention depends on the spatial proximity to distractors - a point that could also be pertinent to the findings here.

Moreover, the tracing of a "solution" trail in a maze may be spontaneous and not only a top-down voluntary operation (Wong & Scholl, 2024), a finding that requires a more careful framing of the link to conscious perception discussed in the manuscript.

Conceptualizing attention as a spatial spotlight may therefore oversimplify its role in navigation and planning. Perhaps the observed attentional modulation reflects a perceptual stage of building the trail in the maze rather than a filter for a later representation for more efficient decision making and planning. A fuller discussion of whether the current model and data can distinguish between these frameworks would benefit readers.

We thank the reviewer for highlighting relevant findings in the attention literature that were missing from our discussion. We fully agree that a complete account of the interplay of planning, navigation, and attention is likely to recruit the kind of curvetracing processes highlighted by the reviewer. However, we emphasise that our current focus is not on the process of navigation through a maze, but on the process of construing the maze itself. In other words, we are focused not on how people represent their path from A to B, but how they represent the maze itself, which they then use as a basis for planning between A and B. The VGC model predicts that a subset of obstacles will be included in this construal. We think that a spotlight model is a good starting point for this work, because attention is being deployed across the whole maze stimulus, and then becomes attached to particular objects located in particular positions. This is a distinct process from that involved in navigating the path itself. Accordingly, our stimuli were designed such that task-relevant obstacles could be presented either proximally or distally to the optimal path (e.g., Figure 1a and Supplemental Figures S1-6). An obstacle that blocks any possible path on one side of the maze is task-relevant but located a long way from the optimal path. The results of Ho and colleagues’ (2022) third experiment demonstrate how task-relevant yet distal obstacles are better remembered than task-irrelevant proximal obstacles (see Figure 4 of Ho et al., 2022). We also observed that obstacles further away from the navigation path were often represented by participants (see Figures S1-6), which cannot be explained by curve tracing alone.

While these results cannot definitively rule out the possibility that participants automatically trace the path while also construing the maze, they suggest that the value-guided construal process is an independent predictor of participants’ representations beyond proximity to the navigated path. To make this distinction clearer, we now cite the papers alluded to by the reviewer, in the Discussion on pages 28-29, while also acknowledging the potential for investigating attention during the navigation process itself:

“Future work may also wish to examine the relevance of visuospatial attention for the navigation process itself in this task. While our present findings speak to how individuals perceive the maze while planning, it remains unclear how attention is deployed during navigation along a path, such as how object-based attention progressively spreads along trajectories in time and space(Pooresmaeili & Roelfsema, 2014; Wong & Scholl, 2024).”

There is also one additional nuance to the current spotlight model that we were inspired to consider by the reviewer’s comment. This is the idea that attentional effects may spread within or along the obstacles themselves. We cannot explore this in the current data because we asked for awareness of the entire obstacles, not parts of obstacles, but it may be possible to explore this in future work, for instance, with eye tracking measures.

More generally, the growth-cone (i.e., zoom lens) model of attention for curve tracing proposed by Roelfsema and colleagues shares considerable similarities with the spotlight of attention model. Both models argue for the grouping of spatially proximal items based on attention. While the growth-cone model argues for varying sizes of zoom lenses (i.e., receptive fields of neurons) that facilitate the tracing of proximal items, both models predict that spatially proximal items are preferentially processed together because of attention. Indeed, the spotlight model could model these varying zoom lenses by altering the width of the attentional spotlight dynamically across the visual scene based on the spatial proximity of obstacles. Following related comments by Reviewer 2, we now investigate inter-individual differences in the attentional spotlight of participants and observed that these differences significantly predict participants’ mental representations (see Attentional spotlight model of task representations). We have now updated the Discussion to include consideration of these alternative model frameworks:

On page 27:

“Second, in the current work we were unable to distinguish whether these attentional effects are driven by a fixed spotlight of attention, or whether attention operates akin to a zoom lens, shifting the ‘width’ of the focus of attention according to the task demands (Eriksen & St. James, 1986; Müller et al., 2003; Schad & Engbert, 2012). The latter view would be consistent with growth-cone models of attention in which the focus of attention expands and contracts in accordance with task demands, mirroring the various receptive field sizes in the visual hierarchy (Pooresmaeili et al., 2014; Pooresmaeili & Roelfsema, 2014). In partial support of this idea, we found significant inter-individual differences in the width of participants’ attentional spotlight (Figure S11). It is also possible that attention is deployed within or along parts of obstacles, rather than on entire obstacles. Future work using naturalistic measures of eye movements may be able to address these questions.”

(3) Lateralization of attention:

The analysis considers whether relevant information is distributed bilaterally or unilaterally across the visual display, but does not sufficiently address evidence for attentional asymmetries across the left and right visual fields due to hemispheric specialization (e.g., Bartolomeo & Seidel Malkinson, 2019). Whether effects differ for left versus right hemifield arrangements is not made explicit in the presented findings.

We thank the reviewer for this suggestion. To address this point, we fitted a three-way interaction model between VGC model prediction, lateralization index, and side (left vs right hemifield). We did not find evidence for the three-way effect (β = 0.01, SE = 0.02, 95% CI [-0.03, 0.04], p = 0.738; ΔBIC = 58.30 in favour of the null effect; see table below), suggesting that the side to which participants lateralized their attention did not influence their task representations. This result is now reported on page 12:

“This effect did not vary significantly as a function of the specific hemifield (i.e., left vs right) in which task-relevant information was presented (β = 0.01, SE = 0.02, 95% CI [-0.03, 0.04], p = 0.738; ΔBIC = 58.30 in favour of the null effect; see table S14).”

We also explored inter-individual differences in participants’ tendency to lateralize their attention (see also the next point). We observed that participants tended to lateralize their attention slightly more to the right-hand side for non-lateralized maze stimuli, despite the normative sVGC model predicting that participants should not lateralize their attention for these stimuli (Figure 3c). These results may speak to potential asymmetries in lateralization, but given the exploratory nature of these analyses, they should be verified and replicated in future work.

(4) Individual differences:

Individual differences in attentional modulation are a strength of the work, but similar analyses exploring individual variation in lateralization effects could provide further insight, and the lack of such analyses may mask important effects.

Thank you for this suggestion. In new analyses, we explored whether (i) participants exhibited differences in their tendency to lateralize their awareness reports, and (ii) whether the degree to which they tended to lateralize their awareness predicted their performance on a separate set of maze stimuli. In short, we observed substantial variation in participants’ tendency to lateralize their awareness (Figure S11) and found that this tendency reflected an inter-individual difference which was stable across maze types. We report these new findings on pages 14-16.

“Inter-individual variation in lateralization of attention

Next, we investigated participants’ tendency to pay attention to obstacles within a single hemifield (left vs right) regardless of the sVGC model predictions. To do so, we computed an awareness lateralization index (ALI) based on participants’ self-reported awareness reports of obstacles on each trial (Figure 3a). Large positive values indicate that participants were preferentially aware of the right hemifield, whereas negative values indicate preferential awareness of the left hemifield. Values close to zero indicate that participants paid attention to both hemifields equally (see Methods for details). We observed that participants’ tendency to lateralize their awareness varied greatly across the Ho datasets 1 and 2 (Figure 3b); some participants preferentially paid attention to a single hemifield, regardless of whether the sVGC model predictions were lateralized. For the dSC1 dataset, we observed that on some trials, participants significantly lateralized their awareness (|ALI| > 0.5; Figure 3c) even though the sVGC model predictions were non-lateralized. These findings suggest that participants’ tendency to pay attention to a single hemifield may represent an observable inter-individual difference in how they allocate their awareness to form task construals.”

“To further explore these inter-individual differences, we tested whether participants’ tendencies to lateralize their attention to a single hemifield was consistent across trials and maze stimuli. We observed that participants’ tendency to lateralize their attention to a single hemifield was similar for left and right lateralized maze stimuli (Spearman ⍴ = 0.72, Figure 3d). This suggests that participants who preferentially attended to a single hemifield did so regardless of which hemifield they should attend to. More consequentially, the tendency for participants to lateralize their awareness on maze stimuli whose model predictions were also lateralized linearly correlated with participants’ tendency to lateralize their attention on non-lateralized maze stimuli (Spearman ⍴ = 0.88, Figure 3d). Taken together, these findings emphasize that some individuals tend to preferentially attend to a single hemifield when planning. This tendency, importantly, represents an inter-individual difference in how participants allocate their attention across various maze types.”

(5) Distinction between overt and covert attention:

The current report at times equates eye movement patterns with the locus of attention. However, attention can be covertly shifted without corresponding gaze changes (see, for example, Pooresmaeili & Roelfsema, 2014).

We fully agree, and thank the reviewer for prompting further reflection on this distinction. In the online experiments run by Ho and colleagues (i.e., datasets Ho1 and Ho2), participants’ eye movements were not tracked, and therefore, they could not disambiguate whether participants were engaging in covert or overt attention to sample maze obstacles. In our third experiment (i.e., dataset dSC1), we both recorded eye movements and explicitly instructed participants to fixate centrally while viewing the maze. This ensured that participants oriented their attention only covertly during planning (see Figure S13-14).

We now elaborate on this important distinction in the Results section of the manuscript, page 12:

“In addition, we monitored participants’ eye movements in dataset dSC 1 to ensure that attention shifts would be covert as opposed to overt—a distinction which could not be determined in the online samples of datasets Ho 1 and 2.”

On page 28:

“Importantly, while the visuospatial attention effects observed in the Ho 1 and 2 datasets are likely driven by both covert and overt shifts in attention, the findings presented in experiment 3 (i.e., dSC1 dataset) rule out the contribution of overt shifts in attention through the use of eye tracking (see Figure S13-14)(Carrasco, 2011; Pooresmaeili & Roelfsema, 2014).”

The implications for interpreting the relationship between eye movement, memory, and attention in this setting are not fully addressed. The potential dynamics of attention along a maze trajectory and their impact on lateralization analysis would benefit from further clarification.

We thank the reviewer for urging more clarity here. The attentional dynamics we document in our study concern how people perceive / construe the maze itself, rather than how they deploy their attention to guide active navigation. We have now sought to make this distinction clear at a number of points in the paper. The core idea is that attention acts as an early filter to select which obstacles are part of a task construal, which then affects both awareness and memory.

We have now clarified the focus of our study in the introduction on pages 5-7:

“Our focus in this study was to examine how participants perceive and represent their environment (the maze stimulus). This is a distinct process to how participants orient their attention during navigation itself, which is not part of our current study. To do so, we harness classical signatures of attentional selection to characterise how visuospatial attention shapes awareness of maze obstacles during planning.”… “Our focus in the present study was to examine attentional effects on participants’ perception of the maze stimulus. We did not quantify how individuals deploy their attention in the phase in which they were navigating through the maze.”

We did not explicitly test for memory effects in our new experiments, but Ho and colleagues demonstrated that the sVGC model predicted not only awareness reports, but also participants’ memory of obstacles (see Ho et al., 2022). Indeed, task representations computed from memory or awareness reports were strikingly similar in their experiments (Spearman ⍴ = 0.86 between memory accuracy and awareness; ⍴ = 0.86 between confidence in memory and awareness). In relation to eye movements, we refer the reviewer back to our previous response, which details how eye movements were measured and controlled during maze construal.

Figure 1 legend (b) --> (c)

We have corrected this typo in the figure caption.

**Reviewer #2 (Public review):**

Summary:

Castanheira et al. investigate the role of spatial attention for planning during three maze navigation experiments (one new experiment and two existing datasets). Effective planning in complex situations requires the construction of simplified representations of the task at hand. The authors find that these mental representations (as assessed by conscious awareness) of a given stimulus are influenced by (spatially) surrounding stimuli. Individual participants varied in the degree to which attention influenced their task representations, and this attentional effect correlated with the sparsity of representations (as measured by the range of awareness reports across all stimuli). Spatially grouping taskrelevant information on either the left or right side of the maze led to mental representations more similar to optimal representations predicted by the valueguided construal (VGC) model - a normative model describing a theoretical approach to simplifying complex task information. Finally, the authors propose an update to this model, incorporating an attentional spotlight component; the revised descriptive model predicts empirical task representations better than the original (normative) VGC model.

Strengths:

The novelty of this study lies in the proposal and investigation of a cognitive mechanism through which a normative model like value-guided construal can enable human planning. After proposing attention as this mechanism, the authors make concrete hypotheses about mismatches between the VGC predictions and real human behavior, which are experimentally validated. Thus, not only does this study describe a possible mechanism for simplification of task information for planning, but the authors also propose a descriptive model, revising VGC to incorporate this attentional component.

A strength of this paper is the variety of investigative approaches: analysis of existing data, novel experiment, and a computational approach to predict experimental findings from a theoretical model. Analyzing pre-existing datasets increases the size of the participant cohort and strengthens the authors' conclusions. Meanwhile, comparing the predictions of the existing normative model and the authors' own refined model is a clever approach to substantiate their claims. In addition, the authors describe several crucial controls, which are key to the interpretability of their results. In particular, the eye tracking results were critical.

In summary, this paper constitutes an important step toward a more complete understanding of the human ability to plan.

We thank the Reviewer for their thoughtful and positive assessment of our findings. We also appreciate the constructive feedback on our methodology, which we believe has substantially improved our manuscript.

Weaknesses:

(1) There is a critical conceptual gap in the study and its interpretation, mainly due to the reliance on a self-report metric of awareness (rather than an objective measure of behavioral performance).

a. Awareness is tested by a 9-point self-report scale. It is currently unclear why awareness of task-irrelevant obstacles in this task would necessarily compromise optimal planning. There is no indication of whether self-reported awareness affects performance (e.g., navigation path distance, time to complete the maze, number of errors). Such behavioral evidence of planning would be more compelling.

We thank the reviewer for prompting further reflection on the connection between construal and navigation performance. We wish to emphasise that the primary focus of our study was on measuring and modeling participants’ task construals using perceptual awareness judgments, building on the methods developed by Ho and colleagues, rather than on navigation performance itself. However, as the reviewer points out, there is a natural relationship between construal and performance – if you represent the wrong obstacles, plans may be disrupted.

To explore the relationship between task construals and performance on the navigation task we first regressed out the effects of the sVGC model on participants’ awareness reports and computed the mean squared residuals for each trial. We then used these values to predict participants’ navigation response times on each trial. We observed a significant negative relationship, suggesting that on trials where participants’ representations showed greater deviations from the normative model, they were in fact faster at navigating the mazes. This relationship was surprising, and at odds with the initial idea that adhering to normative VGC aids in task performance. However, we think that this direction of effect may make sense if one considers that a large part of the actual construal (rather than the normative prediction) in our data was in fact driven by effects such as lateralisation which are not accounted for by the sVGC model. If one is faster at harnessing inductive biases such as lateralisation, then one may be faster to complete the maze but also show a greater deviation from the predictions of the original model.

To further explore these effects, we next focused on the distinction between lateralised and non-lateralised mazes. Here, we reasoned that the initial phase of lateralised attentional selection would lead to lateralised mazes being easier to navigate than nonlateralised ones. We conducted new analyses to determine whether participants navigated lateralized maze stimuli faster and with fewer moves than maze stimuli with non-lateralized model predictions. As detailed in Methods, we excluded trials in which participants significantly deviated from the optimal number of moves (9 or more moves) and took longer than 20 seconds to solve the maze. In line with our interpretation that attention operates as an inductive bias, participants were faster and deviated less from the optimal path on lateralized compared to non-lateralized mazes.

We now report these new results on navigation performance on pages 20-21:

“Maze navigation performance

The previous analyses focused on participants’ task representations during planning. We next sought to explore links between participants’ task representations and maze navigation performance. Participants performed the maze navigation task near-ceiling: they solved 95% of maze stimuli in under 20 seconds, with minimal deviation from the optimal path (i.e., 9 moves or fewer). Notwithstanding this limited variance in task performance, we explored whether participants’ task construals may have impacted their navigation speed. To do so, we first regressed out the effects of the sVGC model from participants’ awareness reports and used the mean squared residuals for each trial to predict response times (see Methods for details). Surprisingly, we observed a negative relationship between mean squared residual variance and response times (β = -0.31, SE = 0.05, 95% CI [-0.41, -0.21], *p* < 0.001), indicating that participants were faster on trials where the sVGC model explained less variance in their awareness reports. In other words, trials in which participants deviated more from the sVGC model predictions were solved faster. We note that one reason for this may be the strong influence of the lateralisation effect on navigation performance (see paragraph below), which itself is not part of the sVGC model prediction.”

“We then explored whether participant performance differed between lateralised and nonlateralised mazes. Here, we reasoned that the initial phase of lateralised attentional selection would lead to lateralised mazes being easier to navigate than non-lateralised ones. Consistent with this hypothesis, participants were faster (β = -0.04, SE = 5.91*10^3^, 95% CI [-0.06, -0.03], *p*< 0.001) and followed the optimal path more closely (β = -0.59, SE = 0.09, 95% CI [-0.78, -0.40], *p*< 0.001) when maze stimuli were more lateralized.”

And in the Discussion section, on page 23:

“Mental representations and task performance

We observed that participants were faster and deviated less from the optimal path on maze stimuli that were lateralized. This effect is not predicted by the original sVGC model but dovetails with the interpretation that early visuospatial attention operates as an inductive bias to guide the formation of simplified task representations. Surprisingly, we also observed that participants were faster to navigate mazes on trials where their simplified task representation deviated from the sVGC model prediction. We interpret this seemingly contradictory finding in the following way: there are several factors beyond the sVGC model – including, for instance, maze lateralisation – that predict both construal and performance on the maze navigation task. Further work is needed to understand how inductive biases such as lateralisation shape both construal and performance, and the real-world benefits that such strategies might afford for naturalistic stimuli.”

b. Relatedly, it would have been more convincing to have an objective measure of awareness, for instance, how the presence or absence of a "task-irrelevant" obstacle affects performance (e.g., change navigation path distance or time to complete the maze), or whether participants can accurately recall the location of obstacles.

We thank the reviewer for prompting further reflection on the validity and robustness of our awareness measures. We emphasise however that our focus is not (primarily) on maze navigation performance, but on task construal, which as noted in our previous response may come apart from navigation performance for a variety of reasons. Our primary goal is to measure participants’ subjective awareness of the maze as a marker of their idiosyncratic (conscious) mental representation on each trial. In doing so, we build on a rich tradition of measuring subjective awareness in consciousness and perception science (for instance, work using the Perceptual Awareness Scale, or detection judgments). In this sense, we think our awareness scale (following Ho et al.) represents a valid and straightforward way of assessing our target psychological construct. However, we also agree with the Reviewer that convergent evidence from other measures is always valuable. In Ho and colleagues’ original paper, they developed a variant of the maze task where participants had to recall the location of obstacles, as well as rate their awareness (Exp 3) and a variant in which participants could hover their mouse over hidden obstacles in the maze to reveal their location – an online metric of attentional deployment (Exp 4). These data afforded us the opportunity to validate the awareness reports against an objective measure of recall, as suggested by the Reviewer. In reanalysing these data, we observed that the obstacle awareness and memory/hover measures were strikingly correlated within two independent samples of participants (Spearman ⍴ = 0.86 between memory accuracy and awareness; ⍴ = 0.86 between confidence in memory and awareness; ⍴ = 0.76 between the probability of hovering over the obstacle and awareness; ⍴ = 0.65 between the duration of the mouse hovering and awareness). These re-analyses are now reported on page 22 of our manuscript, to highlight the convergent validity of the awareness metric:

“Finally, we examined the convergent validity of participants’ awareness reports by reanalyzing the memory recall data reported in Ho and colleagues’ experiment(Ho et al., 2022). We reasoned that participants should demonstrate similar task representations regardless of the measure used to probe the construal. In line with this prediction, we observed that the obstacle awareness reports and memory/hover measures were strikingly correlated within three independent samples of participants (Spearman ⍴ = 0.86 between memory accuracy and awareness; ⍴ = 0.86 between confidence in memory and awareness; ⍴ = 0.76 between the probability of hovering over the obstacle and awareness; ⍴ = 0.65 between the duration of the mouse hovering and awareness; see Tables S18 and S19).”

c. Consequently, I'm not sure that we can conclude that the spatial context does impact participants' ability to plan spatial navigation or to "incorporate taskrelevant information into their construal". We know that the spatial context affects subjective (self-reported) awareness, but the authors do not present evidence that spatial context affects behavioral performance.

Following the line of argument above, we think it’s important to separate out task construal (the simplified representation of the maze, measured by awareness reports), and the impact of this on navigation and other aspects of behaviour. The awareness reports (and other convergent measures) show that task-relevant information (as predicted by the VGC) is incorporated into the construal, a process which is modulated by spatial context. These are the key targets of our modeling. Whether this impacts performance is a distinct question, and one that we now address in our response to point a above.

d. Another concern that may complicate interpretation is the following: Figure 3c shows improved VGC model predictions (steeper slope) for mazes with greater lateralization. However, there are notable outliers in these plots, where a high lateralization index does not correspond to good model performance. There is currently no discussion/explanation of these cases.

The Reviewer astutely points out some outliers in our analysis. While on average lateralized maze stimuli are represented more closely to the sVGC model, there are indeed some noticeable outlier mazes. These mazes represent stimuli in which participants tended to lateralize their attention to the ‘wrong hemifield’—e.g., participants were more aware of obstacles in the right hemifield despite sVGC model predicting that obstacles on the left hemifield were task-relevant. We believe this explains the poor sVGC model fits on these trials. We note, however, that on average participants were capable of attending to the correct hemifield without explicit instructions (i.e., 9 out of 12 mazes).

We have now included a discussion of these outliers in the results section of the paper on page 12:

“We note that for three maze stimuli whose model predictions were lateralized there was nevertheless a poor fit to the sVGC model (see Figure 2c, right panel). These outliers correspond to maze stimuli where participants, on average, lateralized their attention to the incorrect hemifield (i.e., the opposite hemifield to that predicted by the sVGC model).”

(2) I noticed an issue with clarity regarding task-relevance. It is currently not fully clear which obstacles are "task irrelevant". Also, the term is used inconsistently, sometimes conflating with "awareness". For example, in the "Attentional spotlight model of task representations" section, the authors state that "taskrelevant information becomes less relevant when surrounded by task-irrelevant information". But they really mean that participants become less aware of those task-relevant obstacles. I assume task-relevance is an objective characteristic related to maze organization, not to a participant's construal. Indeed, the following paragraph provides evidence of model predictions of awareness.

We apologize for any confusion regarding the terminology of our manuscript. We indeed use the terms task-relevant and task-irrelevant to refer to obstacles that are objectively predicted by the normative sVGC model or the attentional spotlight model to be included in (>0.5) or excluded from (<0.5) task construals, respectively. This designation reflects the predictions from the computational model and does not reflect participants’ reported awareness. We then ran linear hierarchical models to predict participants’ awareness reports from these model predictions. The Reviewer is correct that the task-relevance of obstacles is indeed related to the maze’s organization, and not related to participants’ subjective reports of awareness. We have now clarified this point throughout the manuscript to better emphasize the difference between the model predictions of taskrelevance and participants’ subjective reports.

On page 17:

“To achieve this, we computed the predictions of the existing VGC model for each obstacle’s task relevance in a given maze, and averaged these predictions within an attentional spotlight of 3 squares (Figure 4a & S8, see Methods for details). This process yielded novel model predictions, whereby some obstacles which were once predicted as task-irrelevant by the normative sVGC are now predicted as task-relevant by the attentional spotlight model. We depict the effects of this spatial spotlight in Figure 4a: task-irrelevant stimuli (plotted in grey; see middle left obstacle) neighbouring taskrelevant obstacles (plotted in orange) become more task-relevant, whereas taskrelevant information becomes less relevant when surrounded by task-irrelevant information (see bottom right orange obstacle). This deviation in model predictions from the normative sVGC model was used to predict participants’ awareness reports. We hypothesized that this spotlight-VGC model would predict participants’ reports better than the original VGC model, which does not account for spatial attention.”

(3) The behavioral paradigm has some distinct disadvantages, and the validity of the task is not backed up by behavioral data.

a. I understand the need for central fixation, but it also makes the task less naturalistic.

The fixation cross was required on every trial such that participants could maintain central fixation for our eye tracking experiment. While this design is less naturalistic, it allows us to examine the eye movements of participants. Requiring participants to fixate during the ‘planning’ phase of the experiment allowed us to isolate the effects of covert attention from changes in awareness due to overt shifts in attention. In other words, differences in participants’ awareness reports in the 3rd experiment cannot be explained by longer fixation times to specific obstacles.

b. The task with its top-down grid view does not seem to mimic real human navigation. Though this grid may be similar to mental maps we form for navigation, the sensory stimuli corresponding to possible paths and to spatial context during real-life navigation are very different.

We agree with the reviewer that while our task is engaging for participants and simple to follow, it does not mimic naturalistic navigation in humans. There is a natural tension in computational / experimental work in cognitive science in wanting to build closely on previous results and paradigms, while ensuring that results can generalise to real-world contexts. Here, our choice of paradigm and measures was closely built on previous papers using this task from Ho and colleagues (2022, 2023). While preparing this response, we learnt that the MIT group had also harnessed this same task to develop a novel dynamic variant of the VGC model (Chen et al., 2026) called the Just in Time model (JIT). The advantage of building on this prior work is that we are able to iteratively refine and expand the VGC approach, and (in our case) bring it into closer contact with work on modeling the deployment of spatial attention in human vision. The top-down aspect of the maze notably facilitated the study of the spatial deployment of attention. We now discuss the novel dynamic variant of the VGC model in our paper on page 27:

“We close by reflecting on opportunities for further work in this area. First, an important next step is to explore the process by which task representations are formed, and how inductive biases might affect the process of task construal. The sVGC model is a normative model of the optimal task representation. Since it’s construction involves an exhaustive calculation over possible paths, it is not a plausible basis for a model of the psychological process by which participants actually construct task representations. More recently a process model of task construal has been proposed, the Just in Time model (JIT). The hypothesis of the JIT model is that participants’ task representations are built up over time by iteratively simulating possible paths through the maze, affording insight into the construal process (Chen et al., 2026). In future work, it would be of interest to ask whether the attentional effects we observe in our experiments could be meshed with a dynamic JIT account of construal. We speculate that visuospatial attention may operate as an early filter, limiting the space of potential construals based on coarse spatial features of the environment, constraining a dynamic selection of obstacles. Brain imaging techniques with high time resolution, such as M/EEG, may be able to shed further light on how task representations are formed as participants plan.”

c. Behavioral performance is not reported, so it is unknown whether participants are able to properly complete the task. The task seems pretty difficult to navigate, especially when the obstacles disappear, and in combination with the central fixation.

Behavioural performance is now reported in response to point 1a above.

d. There is no discussion of whether/how this navigation task generalizes to other forms of planning.

We fully agree that an important next step would be to generalise our results on construal to naturalistic forms of planning – for instance, using immersive VR mazes, and or investigating cognitive rather than perceptual construals. We have now added a line to this effect to the Discussion on page 28.

“An important next step to further our understanding of task representations would be to extend the current paradigm to other forms of planning and more naturalistic tasks, such as navigating immersive virtual reality (VR) environments, planning over cognitive rather than perceptual representations (e.g. planning over an abstract space), or internallyguided planning based on working memory.”

**Reviewer #2 (Recommendations for the authors):**

(1) There are, of course, benefits to simple tasks like the ones described, but it would be interesting to compare the results to a possible experiment in which a top-down grid/map is used for planning, but then task execution is carried out in a simulated environment corresponding to the map. Also, perhaps beyond the scope of the questions addressed in this paper, but I am curious how unexpected obstacles affect representations. For instance, if participants plan based on a topdown map and then begin "real" navigation but encounter an unexpected obstacle that was not indicated on the map, does this modulate representations/awareness of future obstacles (near vs. far)?

We fully agree that all of these lines of investigation would be super interesting to pursue in future studies, and we have added a line to the discussion to that effect on page 28:

“An important next step to further our understanding of task representations would be to extend the current paradigm to other forms of planning and more naturalistic tasks, such as navigating immersive virtual reality (VR) environments, planning over cognitive rather than perceptual representations (e.g.. planning over an abstract space), or internallyguided planning based on working memory.”

(2) Regarding self-reported awareness as a metric, an additional experiment could ask participants to recreate the maze (identify locations of obstacles after they disappear). This would be a more objective measure of awareness.

Yes indeed, and as described above, this was a metric used by Ho and colleagues in their previous experiment. As we describe in more detail above, the task representations obtained via memory or awareness reports demonstrated striking similarity (⍴ = 0.86).

(3) What is meant by "all possible orientations of the maze" in this Methods sentence: "For dataset dSC 1, participants solved each of these 24 mazes four times (i.e., all possible orientations of the maze)"?

We thank the Reviewer for prompting more clarity here. We vertically and horizontally reversed mazes (i.e., left-right flipped) such that participants could not predict the location of the goal or start location. In this way, each maze stimulus had four potential orientations. This resulted in 96 trials of 24 unique mazes. We have clarified this point in the Methods section on page 30:

Maze stimuli were vertically and horizontally reversed (i.e., left-right flipped) such that participants could not predict the location of the start or goal location. This resulted in four potential orientations of each maze across all 24 mazes, 96 trials in total.

(4) For lateralization, it was unclear until reading the Methods that the lateralization index was calculated using the VGC-predicted level of taskrelevance. From the main text and Figure 2, I assumed you were just counting the number of task-relevant obstacles on each side, rather than also quantifying relevance. I understood after reading the Methods, but this could be clarified further.

We agree with the Reviewer that this was not evident from the text. We have now updated the Results section of the manuscript to clarify this point on page 11:

“To test this hypothesis, we derived a measure of task-relevant lateralization inspired by the attention literature (Ghafari et al., 2024; Keefe & Störmer, 2021; Vollebregt et al., 2015) (Figure 2a). Specifically, we separated maze stimuli across the vertical meridian and computed the ratio of task-relevant information presented on the left versus right side derived from the sVGC model. For example, the maze shown in Figure 2a has twice the amount of task-relevant information presented in the left hemifield than in the right (lat. Index = 1/3). A lateralization index of 0.0 indicates that both hemifields contain equal amounts of task-relevant information (i.e., non-lateralized). The lateralization index was computed using the continuous VGC predictions for each obstacle (see Methods).”

(5) The explanation in the Methods of how the width of the attentional spotlight was chosen references Figure 1b and Supplementary Figure S2, but it seems that Supplementary Figure S8 explains this more in the caption. Also, I don't see how Figure S2 supports this.

We apologize for this typo. The explanation of how we selected the width of the attentional spotlight should indeed reference supplemental Figure 15 (previously Figure S8). We have now corrected this and elaborated on this choice in the Methods section on page 35:

“We fixed the ‘width’ of the attentional spotlight to a distance of 3 squares based on the observation that the two neighbouring obstacles positively predicted the awareness of a probe. We observed that the mean and median distance between neighbouring obstacles of the 2nd rank (i.e., second closest) was 3 squares away for all mazes (Figure S15). We therefore opted to fix the value of the attention spotlight to 3 squares based on these observations. Future work utilizing this model should consider the statistics of their maze stimuli when deciding on the ‘width’ of the attentional spotlight.”

(6) The attentional spotlight width was assumed to be 3 squares, based on the linear regression predictions of the effect of neighboring obstacles on stimulus awareness. Given the individual differences across participants, it would be interesting to choose a different attentional spotlight size for each participant. Would a participant-specific attentional spotlight width improve the predictions of the spotlight-VGC model?

The Reviewer highlights a very interesting question: do individuals vary in terms of their attentional spotlight? To test this hypothesis, we first estimated the size of the attentional spotlight for each individual based on lateralized maze stimuli, and then used this to generate personalized attentional spotlight model predictions for each subject based on these values (Figure S11). We restricted this analysis to the dSC1 dataset, where we had substantially more trials (96 in total).

In brief, we observed that indeed the personalized spotlight model fit participants’ awareness reports better than both a normative sVGC model and a group-level attentional spotlight model. We interpret these findings with some caution as (i) a subset of individuals had flat attentional slopes and therefore were excluded from these analyses, and (ii) we believe we require additional trials to ensure a robust model fit at the individual level. While our results are encouraging, we hope future investigations into inter-individual differences will extend these findings.

We have included these additional analyses in the main text.

On page 18:

“To further explore inter-individual differences in task construal, we tested whether adjusting the attentional spotlight width to each participant’s awareness reports improved the predictions of the attentional spotlight model. To do so, we first determined the width attentional spotlight of each individual in the dSC1 dataset based on lateralized maze stimuli. We then generated person-specific attentional spotlight model predictions for the non-lateralized maze stimuli to avoid overfitting the data (Figure S11). We note that 7 participants had either flat attentional slopes or negative beta coefficients, which prevented the selection of an appropriate attentional spotlight width (see Methods for details). We observed a significant improvement in model fit for the person-specific attentional spotlight model relative to both the group-level attentional spotlight model (ΔBIC = -1487.39) and the normative sVGC model (ΔBIC = -1655.29). While the limited trial numbers per participant in our current dataset warrants caution in interpreting these findings, these findings do encourage further research on inter-individual differences in attentional deployment during planning.”

On pages 23-24:

“Inter-individual differences in attention

We also observed considerable inter-individual differences in attentional effects across participants (Figure 1c). While some participants were strongly influenced by the spatial context of neighbouring stimuli, others showed more limited evidence for an attentional effect (Figure 1b). Inter-individual differences in attention predicted the sparsity of participants’ simplified representations: participants with larger attention effects exhibited sparser representations. Moreover, these inter-individual differences in effects of spatial proximity could be incorporated into the attentional spotlight model by varying the width of the spotlight, resulting in better model predictions.”

“Beyond these spatial proximity effects, we also observed that participants varied in their tendency to lateralize their attention to a single hemifield (Figure 3). This tendency was observed across all three datasets, including on maze stimuli whose value-guided model predictions were not lateralized. This suggests that although a strategy of allocating attention is sub-optimal for these maze stimuli, some individuals preferentially attend to a single hemifield in a heuristic-like fashion. This tendency to attend to a single hemifield was a robust inter-individual difference across maze stimuli (Figure 3d), and dovetails with individual-level variation in spatial proximity effects. Taken together, these findings offer novel insights into how people vary in the ways they allocate spatial attention to solve complex problems. Future research could explore how these individual differences constrain performance on other tasks that require planning and search in highdimensional spaces.”

On page 17 of the Supplemental Materials:

(7) The supplementary text about lateralization effects, above Supplementary Table S8, references Table S6, but it is Table S6 does not seem to display lateralization results.

We thank the Reviewer for pointing out this typo: we now refer to the correct supplementary table (S9).

(8) Why does it matter that "the maze stimuli were not designed to test horizontalmeridian lateralization effects"? What is the effect on power? Is it because there is not a good enough range in lateralization indices? It would be good to clarify, or just remove that explanation, since the cortical retinotopy explanation seems more convincing.

We did not specifically design the maze stimuli such that there is an equal number of obstacles above and below the horizontal meridian. As such, the lateralization index derived along the horizontal meridian does not control for the number of obstacles in each hemifield, which may influence participants’ awareness reports. In contrast, we designed maze stimuli such that this would not be a concern for the vertical meridian. We have clarified this point in the discussion on page 27.

“Third, while we observed clear lateralization effects along the vertical meridian (i.e., left vs right hemifield), effects along the horizontal meridian were less clear (i.e., above vs below; see Table S15-16). One potential explanation of this asymmetry is the retinotopic organization of the cortex, in which spatially adjacent stimuli can be retinotopically distant if presented on the opposite side of the vertical (but not horizontal) meridian, facilitating distractor inhibition. Importantly, while the visuospatial attention effects observed in the Ho 1 and 2 datasets are likely driven by both covert and overt shifts in attention, the findings presented in experiment 3 (i.e., dSC1 dataset) rule out the contribution of overt shifts in attention through the use of eye tracking (see Figure S13-14)(Carrasco, 2011; Pooresmaeili & Roelfsema, 2014).”

(9) For Figure 2c, it would be helpful to directly state what each dot and line mean.

We updated the caption of Figure 2c to clarify what we are plotting: each point represents an obstacle, and each line the linear fit for a maze stimulus.

“Each point represents an obstacle in a maze, and each line represents the model fit for that specific maze stimulus.”

(10) Figures and wording imply there is only a single probe obstacle per trial, but methods and model imply that participants are asked to report awareness for every obstacle. This should be clarified.

We apologize for any confusion regarding the methodology of our study. The Reviewer is correct that participants reported their awareness of every obstacle presented on a given trial. We have clarified this in the Results section of the manuscript on page 7:

“Note, participants reported their awareness of every obstacle presented on a given trial.”

We have also updated the caption of Figure 1 to clarify this point:

“Once participants finished navigating the maze, they were asked to report their awareness of every obstacle presented on a given trial in a random order.”

(11) What is the reason for the exclusion of participants (33 for experiment 1 and 26 for experiment 2)?

Participants were excluded from the Ho et al. datasets 1 and 2 based on their preregistered exclusion criteria, as detailed in the Methods section of their paper. In short, trials were excluded if participants took longer than 20 seconds to complete the trial, or if they spent longer than 5 seconds in the initial state. Participants were excluded if less than 80% of trials remained after reaction time exclusions or if they failed 2 out of 3 comprehension checks. We have elaborated on this point in the Methods section on page 31.

“Participants were excluded from analyses based on pre-registered exclusion criteria as detailed in (Ho et al., 2022). In short, participants were excluded if 20% or more of their trials were removed based on reaction times, or if they failed 2 out of 3 comprehension checks.”

(12) The supplemental figures are not referenced in order, and some are not referenced at all; this should be fixed.

We thank the Reviewer for pointing this out and have reorganized our Supplementary materials accordingly.

**Reviewer #3 (Public review):**

Summary:

The authors build on a recent computational model of planning, the "value-guided construal" framework by Ho et al. (2022), which proposes that people plan by constructing simple models of a task, such as by attending to a subset of obstacles in a maze. They analyze both published experimental data and new experimental data from a task in which participants report attention to objects in mazes. The authors find that attention to objects is affected by spatial proximity to other objects (i.e., attentional overspill) as well as whether relevant objects are lateralized to the same hemifield. To account for these results, the authors propose a "spotlight-VGC" model, in which, after calculating attention scores based on the original VGC model, attention to objects is enhanced based on distance. They find that this model better explains participant responses when objects are lateralized to different hemifields. These results demonstrate complex interactions between filtering of task-relevant information and more classical signatures of attentional selection.

Strengths:

(1) The paper builds on existing modeling work in a novel manner and integrates classic results on attention into the computational framework.

(2) The authors report new and extensive analyses of existing data that shed light on additional sources of systematic variability in responses related to attentional spillover effects

(3) They collect new data using new stimuli in the original paradigm that directly test predictions related to the lateralization of task-relevant information, including eye tracking data that allows them to control for possible confounds.

(4) The extended model (spotlight-VGC) provides a formal account of these new results.

We thank the Reviewer for their positive assessment of our manuscript and their insightful comments, which has improved the clarity of our findings.

Weaknesses:

(1) The spotlight-VGC model has a free parameter - the "width" of the attentional spotlight. This seems to have been fixed to be 3 squares. It would be good if the authors could describe a more principled procedure for selecting the width so that others can use the model in other contexts.

Our choice for this parameter was informed by the spatial effects reported in Figure 1b. We observed that the two closest neighbouring obstacles to a probe had similar awareness (i.e., positive beta weights). We therefore compute the mean and median distances between obstacle pairs that were the second closest obstacle to a probe. This distance was 3 squares away, as depicted in Figure S15. We fixed the width of the attentional spotlight across all studies based on this observation. We agree that future research utilizing this model may need to tune this hyperparameter depending on the mean distance between a probe and its neighbours.

We have clarified this point in the methods section on page 35:

“We fixed the ‘width’ of the attentional spotlight to a distance of 3 squares based on the observation that the two neighbouring obstacles positively predicted the awareness of a probe. We observed that the mean and median distance between neighbouring obstacles of the 2nd rank (i.e., second closest) was 3 squares away for all mazes (Figure S15). We therefore opted to fix the value of the attention spotlight to 3 squares based on these observations. Future work utilizing this model should consider the statistics of their maze stimuli when deciding on the ‘width’ of the attentional spotlight.”

Following the suggestion of Reviewer 2 point 6, we now also explored inter-individual differences in this parameter. To do so, we first used the lateralized mazes in the dSC1 dataset to determine the optimal width of the attentional spotlight for each individual.

Then, we used this spotlight to derive model predictions for each person. We observed that these personalized attentional spotlight model predictions fit participants’ awareness reports on non-lateralized mazes better than the fixed-width spotlight model. We believe this preliminary result suggests the importance of modelling inter-individual differences in attentional deployment during planning. We report these effects on page 17.

(2) Have the authors considered other ways in which factors such as attentional spillover and lateralization could be incorporated into the model? The spotlightVGC model, as presented, involves first computing VGC predictions and only afterwards computing spillover. This seems psychologically implausible, since it supposes that the "optimal" representation is first formed and then it gets corrupted. Is there a way to integrate these biases directly into the VGC framework, perhaps as a prior on construals? The authors gesture towards this when they talk about "inductive biases", but this is not formalized.

We thank the reviewer for bringing up this very important point. We think that a full computational treatment of the inductive bias would be a distinct project, but now seek to expand our discussion on the mechanisms by which representations could be formed. In this context, we specifically highlight novel computational work from the MIT group that was published as a preprint in the time since we submitted our paper, and which proposes a new process account of construal, the “Just in Time” (JIT) model. We also elaborate on a possible mechanism by which visuospatial attention may aid the dynamics of the construal process. In short, we agree with the reviewer that spatial attention may bias individuals to search over a subset of potential representations based on low-level spatial characteristics of the obstacles (e.g., their spatial spread in the visual field), prior to (or in concert with) a dynamic JIT-like selection process. We now elaborate on these possibilities on pages 27-28:

“We close by reflecting on opportunities for further work in this area. First, an important next step is to explore the process by which task representations are formed, and how inductive biases might affect the process of task construal. The sVGC model is a normative model of the optimal task representation. Since it’s construction involves an exhaustive calculation over possible paths, it is not a plausible basis for a model of the psychological process by which participants actually construct task representations. More recently a process model of task construal has been proposed, the Just in Time model (JIT). The hypothesis of the JIT model is that participants’ task representations are built up over time by iteratively simulating possible paths through the maze, affording insight into the construal process (Chen et al., 2026). In future work, it would be of interest to ask whether the attentional effects we observe in our experiments could be meshed with a dynamic JIT account of construal. We speculate that visuospatial attention may operate as an early filter, limiting the space of potential construals based on coarse spatial features of the environment, constraining a dynamic selection of obstacles. Brain imaging techniques with high time resolution, such as M/EEG, may be able to shed further light on how task representations are formed as participants plan.”

[…]

“Fourth, it will also be necessary to elaborate on how bottom-up and top-down aspects of attentional selection are combined to guide complex task representations and plans. Foundational questions remain unanswered, for instance: can multiple spatial locations be preferentially selected at once, i.e. are there multiple spotlights (Awh & Pashler, 2000; McMains & Somers, 2004; Pylyshyn & Storm, 1988; Shaw & Shaw, 1977)? There is also discourse on how spatial attention may move from one location to another: are the intervening visual regions between attended locations similarly selected (Dubois et al., 2009; Kr & Np, 1999; McMains & Somers, 2004, 2005)? Our findings tentatively suggest that individuals are able to attend to disparate spatial regions to form sparse task representations, yet there is substantial variability in how individuals orient their attention during the task. The present paradigm and computational modelling, in conjunction with carefully designed stimuli, may help resolve these outstanding questions.”

(3) Can the authors rule out that the lateralization effects are the result of memory biases since the main measure used is a self-report of attention?

We thank the reviewer for bringing up this important point. In our experiments, we sought to measure participants’ subjective awareness of the maze stimuli as a readout of their conscious task representation on each trial. This approach marries an extensive literature on measures of perceptual awareness in consciousness science (e.g., using the Perceptual Awareness Scale) with computational models of planning. Participants’ memory of (their awareness of) the obstacles is inherent to this approach, but just as with similar approaches in consciousness science (e.g. measures of iconic memory in the Sperling paradigm), we think it provides a reasonably “online” measure of awareness. It’s important of course to ensure that results obtained with awareness reports are not idiosyncratic, and generalise to other approaches to quantifying task representations.

To further bolster the convergent validity of our awareness measure, we reanalyzed the data from Ho and colleagues. In their original paper, they developed a variant of the maze-navigation task where participants were asked to recall the location of obstacles as well as report their awareness (Exp 3) and a third variant of the task where participants could hover their cursors over hidden obstacles to reveal their locations (Exp 4). These data allowed us to validate the awareness reports against objective measures of recall and mouse-tracking data. We observed that the subjective awareness reports of participants were strikingly correlated with recall/hover measures across two independent samples of participants (Spearman ⍴ = 0.86 between memory accuracy and awareness; ⍴ = 0.86 between confidence in memory and awareness; ⍴ = 0.76 between the probability of hovering over the obstacle and awareness; ⍴ = 0.65 between the duration of the mouse hovering and awareness). We believe these findings validate participants’ awareness reports. These findings are now reported on page 22 of the manuscript.

“Finally, we examined the convergent validity of participants’ awareness reports by reanalyzing the memory recall data reported in Ho and colleagues’ experiment (Ho et al., 2022). We reasoned that participants should demonstrate similar task representations regardless of the measure used to probe the construal. In line with this prediction, we observed that the obstacle awareness reports and memory/hover measures were strikingly correlated within three independent samples of participants (Spearman ⍴ = 0.86 between memory accuracy and awareness; ⍴ = 0.86 between confidence in memory and awareness; ⍴ = 0.76 between the probability of hovering over the obstacle and awareness; ⍴ = 0.65 between the duration of the mouse hovering and awareness; see Tables S18 and S19).”
